# SUMO Chain Formation Is Required for Response to Replication Arrest in *S. pombe*

**DOI:** 10.1371/journal.pone.0006750

**Published:** 2009-08-25

**Authors:** Andrew Skilton, Jenny C. Y. Ho, Brenda Mercer, Emily Outwin, Felicity Z. Watts

**Affiliations:** Genome Damage and Stability Centre, University of Sussex, Falmer, Brighton, United Kingdom; Universidade de Sao Paulo, Brazil

## Abstract

SUMO is a ubiquitin-like protein that is post-translationally attached to one or more lysine residues on target proteins. Despite having only 18% sequence identity with ubiquitin, SUMO contains the conserved ββαββαβ fold present in ubiquitin. However, SUMO differs from ubiquitin in having an extended N-terminus. In *S. pombe* the N-terminus of SUMO/Pmt3 is significantly longer than those of SUMO in *S. cerevisiae*, human and *Drosophila*. Here we investigate the role of this N-terminal region. We have used two dimensional gel electrophoresis to demonstrate that *S. pombe* SUMO/Pmt3 is phosphorylated, and that this occurs on serine residues at the extreme N-terminus of the protein. Mutation of these residues (in *pmt3-1*) results in a dramatic reduction in both the levels of high Mr SUMO-containing species and of total SUMO/Pmt3, indicating that phosphorylation of SUMO/Pmt3 is required for its stability. Despite the significant reduction in high Mr SUMO-containing species, *pmt3-1* cells do not display an aberrant cell morphology or sensitivity to genotoxins or stress. Additionally, we demonstrate that two lysine residues in the N-terminus of *S. pombe* SUMO/Pmt3 (K14 and K30) can act as acceptor sites for SUMO chain formation *in vitro*. Inability to form SUMO chains results in aberrant cell and nuclear morphologies, including stretched and fragmented chromatin. SUMO chain mutants are sensitive to the DNA synthesis inhibitor, hydroxyurea (HU), but not to other genotoxins, such as UV, MMS or CPT. This implies a role for SUMO chains in the response to replication arrest in *S. pombe*.

## Introduction

Post-translational modification is an efficient and rapid way of controlling the activity of proteins. A variety of species have been identified that can be attached post-translationally to proteins. In many cases, modification involves small species, e.g. in the phosphorylation, acetylation and methylation of proteins, while in others the modifying species are larger, e.g. in the case of ubiquitination and sumoylation, which involve the proteins ubiquitin and SUMO respectively e.g. [1], [2].

SUMO is a member of the Ubl (ubiquitin-like) family of post-translational modifiers. Although it has only 18% sequence identity with ubiquitin, its structure resembles that of ubiquitin, in that it contains the conserved ubiquitin ββαββαβ fold [3], [4]. Ubiquitin comprises 76 aa, while SUMO is larger, having an extended N-terminus (in the order of 15–30 aa) that is not present in ubiquitin. The major role of ubiquitin is in targeting proteins for proteasome-mediated proteolysis (reviewed in [5]). However, ubiquitin also has important roles in modifying the function of individual proteins required for specific processes, e.g. ubiquitination of PCNA (proliferating cell nuclear antigen) is required for bypass of replication blocking lesions in DNA [6], [7], [8]. SUMO modification has a variety of cellular functions, including roles in transcription, DNA damage responses, the cell cycle and nuclear transport e.g. [9], [10], [11]. Recently it has been shown to be required for STUbL- (SUMO-targeted ubiquitin ligase)-dependent ubiquitination of target proteins e.g. [12], [13], [14].

The process of sumoylation resembles that of ubiquitination (reviewed in [15]). Like ubiquitin, SUMO is produced as a precursor protein that needs to be cleaved to the mature form by one or more specific SUMO proteases (Ulps). This processing reveals a GG motif at the C-terminus of SUMO, which is required for its subsequent activation and conjugation to target proteins. Mature SUMO is first activated by a heterodimeric activator protein via the formation of a thioester linkage. It is then transferred to a SUMO conjugator, again forming a thioester link. From here, SUMO is attached to one or more lysine residues on the target protein via an ε−amino bond. In many cases, the acceptor lysine is present within the context ψKxE, where ψ is a bulky hydrophobic amino acid and x is any residue. In some instances attachment of SUMO to target proteins is enhanced by one of relatively few SUMO ligases.

It is well documented that ubiquitin forms chains (e.g. [16]). This can occur through a number of lysine residues within ubiquitin, predominantly K6, K29, K48 and K63. Initial reports on SUMO modification suggested that, unlike ubiquitin, SUMO did not form chains. However, several findings have established that SUMO is capable of forming chains, both *in vitro* and *in vivo* [17], [18], [19], [20]. Despite evidence for their existence *in vivo*, the biological role of SUMO chains is less obvious. An *S. cerevisiae* mutant (*smt3-allR*) in which all potential SUMO acceptor lysines have been mutated to alanine, shows little phenotype during vegetative growth [20]. More recently it has been demonstrated that SUMO chains can interact with STUbLs (via SIMs – SUMO-interacting motifs) [21], implying that they can act as a signal to target proteins for ubiquitin-mediated proteolysis.

The process of sumoylation is generally conserved between eukaryotic organisms. In *S. pombe*, SUMO is encoded by the *pmt3* gene [22]. The ubiquitin-like region of *S. pombe* SUMO/Pmt3 resembles SUMO in other organisms. However its N-terminus is distinctly longer than those in *S. cerevisiae* SUMO/Smt3 or in human SUMO-1/-2/-3 (Figure S1). While SUMO is essential for viability in *S. cerevisiae* and mammals, deletion of the *pmt3* gene is not lethal, although null mutant cells are temperature sensitive for growth and extremely sensitive to a range of toxins [22]. Mutants defective in the *S. pombe* SUMO activator subunit (Rad31), the SUMO conjugator (Hus5) or one of the SUMO ligases, Nse2, are sensitive to DNA damaging agents [23], [24], [25], [26]. In contrast, a null mutant deleted for the *S. pombe* SUMO ligase Pli1 has little phenotype, apart from a mild sensitivity to the microtubule inhibitor thiabendazole (TBZ) [27].

Here we investigate sequence requirements for SUMO/Pmt3 function in *S. pombe*. We show that SUMO/Pmt3 is phosphorylated on serine residues at its extreme N-terminus and that inability to phosphorylate SUMO/Pmt3 results in reduced levels of total SUMO/Pmt3 and reduced levels of high Mr SUMO-containing species *in vivo*. Additionally, we demonstrate that two lysines (K14 and K30) are required for SUMO chain formation, both *in vitro* and *in vivo*. A *pmt3-K14R,K30R* mutant shows cellular abnormalities and sensitivity to HU, indicating that SUMO chain formation is necessary for response to S phase arrest.

## Results

### *S. pombe* SUMO/Pmt3 is phosphorylated

**Figure 1:**
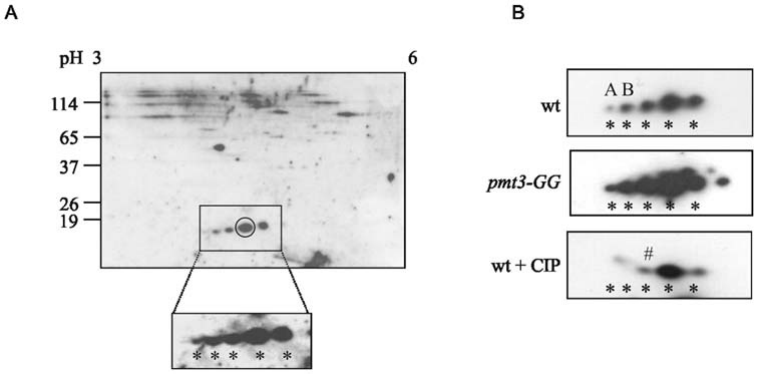
Western analysis of 2D PAGE of *S. pombe* proteins. A. 50 µg of a wild-type total cell extract was separated by IEF (pH 3–6) followed by SDS-PAGE (12.5%) and Western blotted with anti-SUMO antisera. Boxed region is an expanded version of a longer exposure of the same blot. B. Comparison of species in extracts from wt, *pmt3-GG* and wt extracts+CIP (5 units/50 µg protein). * presumed forms of SUMO monomer, A,B, forms not observed after CIP treatment , # possible acetylated form.

As part of our investigation into sumoylated species in *S. pombe* we undertook 2D PAGE. An example of a typical gel (using a low level of protein, 50 µg, and isoelectric focussing range pH 3–6) stained with colloidal Coomassie Blue is shown in Figure S2. Western blotting of a similar 2D gel with anti-SUMO antisera (Figure 1A) showed multiple species, including five which migrate with pIs and Mr similar to that of SUMO/Pmt3. (*S. pombe* SUMO/Pmt3 has a predicted pI of 4.6 and migrates at approximately 18 kDa, e.g. [28]).

One possible explanation for the presence of these species is that they could be intermediates arising during the processing of the precursor form of SUMO/Pmt3 to the mature form. We therefore compared the pattern of species in wild type and *pmt3-GG* cells (where only the mature form of SUMO/Pmt3 is present) (Figure 1B). No difference in the relative amount of the species was observed indicating that the species do not represent processing intermediates.

**Figure 2:**
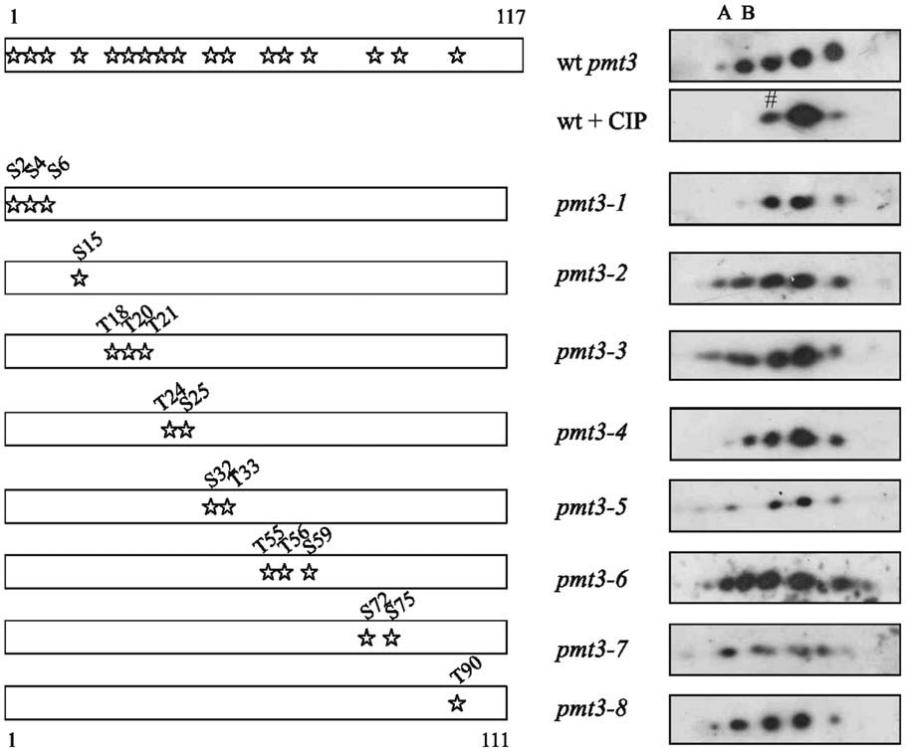
Position of serine and threonine residues and effects of mutations in SUMO/Pmt3. Position of serine and threonine residues in SUMO/Pmt3 are indicated by stars. Left hand panel: sites of mutations in *pmt3* mutants. Right hand panel: Western analysis with anti-SUMO antisera of 2D PAGE (1^st^ dimension pH range 3–6, 2^nd^ dimension 12.5% acrylamide) of extracts from wild type (wt), wild type+CIP and *pmt3* mutants. A,B, forms not observed after CIP treatment, # possible acetylated form.

The pattern of the anti-SUMO antibody cross-reacting species suggests that the species may represent modified forms of SUMO/Pmt3. Since the forms are very similar in size, but have different pIs, it could be postulated that the modifying species is/are small, but charged. One candidate for such a modifying species is phosphate. To determine whether any of these species represent phosphorylated forms, we treated protein extracts with calf intestinal phosphatase (CIP) before analysis by 2D PAGE. Figure 1B and Figure 2 indicate that two prominent acidic forms (A,B) are lost following CIP treatment, consistent with them being phosphorylated forms.

We next analysed the SUMO/Pmt3 sequence for possible phosphorylation sites (Ser or Thr residues). Figure 2 shows that SUMO/Pmt3 has nine Ser residues and eight Thr residues (Figure 2, left hand panel). To identify which of these residues is phosphorylated, we created a series of mutations in *pmt3* (*pmt3-1* - *pmt3-8*) and used 2D PAGE to analyse the pattern of species present in each mutant (Figure 2, right hand panel). In all mutants the Ser or Thr residues were mutated to alanine. As a control we included a wild type extract treated with CIP. Of the eight mutants tested, seven had a similar number of species to that observed in wild type cells. In only one mutant, *pmt3-1* (*pmt3-S2A,S4A,S6A*), did the pattern of species resemble that observed following treatment with CIP. This indicates that phosphorylation occurs at the N-terminus of SUMO/Pmt3, likely on two of the three Ser residues (S2, S4, S6).

**Figure 3:**
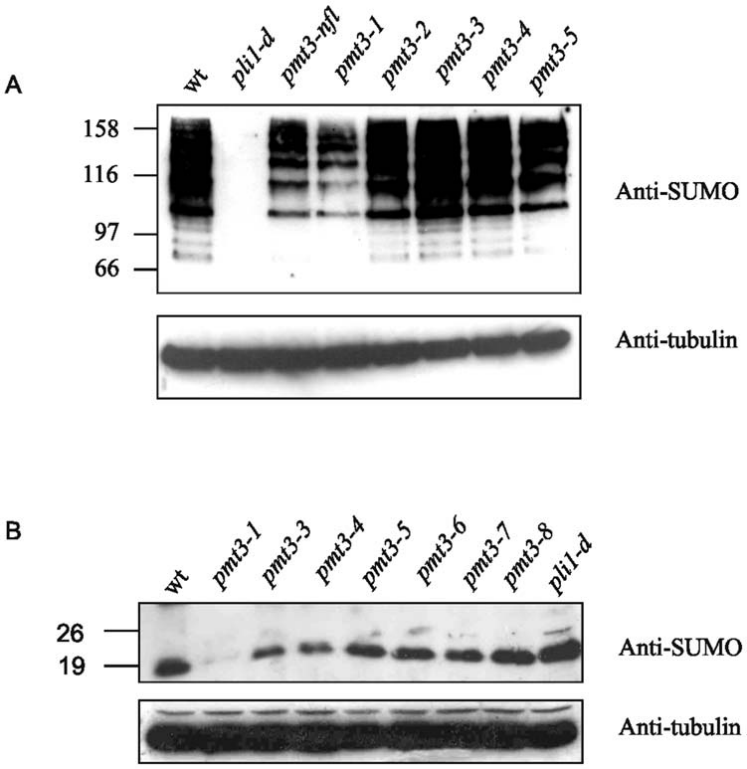
Effect of *pmt3* mutations on levels of sumoylated species and free SUMO/Pmt3 *in vivo*. A. Western blot 7.5% SDS PAG of total cell extracts from wt, *pli1-d* and *pmt3* mutant cells as indicated, probed with anti-SUMO antisera (upper panel) and anti-tubulin antisera (lower panel). B. Western blot 12.5% SDS PAG of total cell extracts from wt, *pli1-d* and *pmt3* mutant cells as indicated, probed with anti-SUMO antisera (upper panel) and anti-tubulin antisera (lower panel).

We next investigated whether inability to phosphorylate the N-terminus of SUMO/Pmt3 affected the levels of high Mr SUMO-containing species in cells. The *pmt3-1* mutant is the only mutant of the eight that we tested that has altered levels of these high Mr species (Figure 3A). In *pmt3-1* the level of high Mr SUMO-containing species is similar to the level observed in *pmt3-nfl* cells (encoding a version of Pmt3 deleted for the first 29 aa), but is still greater than that observed in the SUMO ligase null mutant *pli1-d*.

To determine whether the reduced level of high Mr species in *pmt3-1* cells was due to reduced levels of total SUMO, or reduced ability to incorporate SUMO into chains we compared the levels of free SUMO/Pmt3 in the Ser/Thr mutants and *pli1-d* with that in wild type (Figure 3B and data not shown). Of all the mutants tested, only *pmt3-1* has reduced levels of free SUMO/Pmt3. This was not observed with *pli1-d* cells, where the absence of a SUMO ligase reduces the level of high Mr SUMO-containing species, but not the total amount of SUMO within cells. This suggests that the inability to phosphorylate SUMO/Pmt3 in *pmt3-1* cells affects its stability, and hence the amount of SUMO/Pmt3 available for sumoylation.

### *pmt3-1* is not sensitive to DNA damaging agents

**Figure 4:**
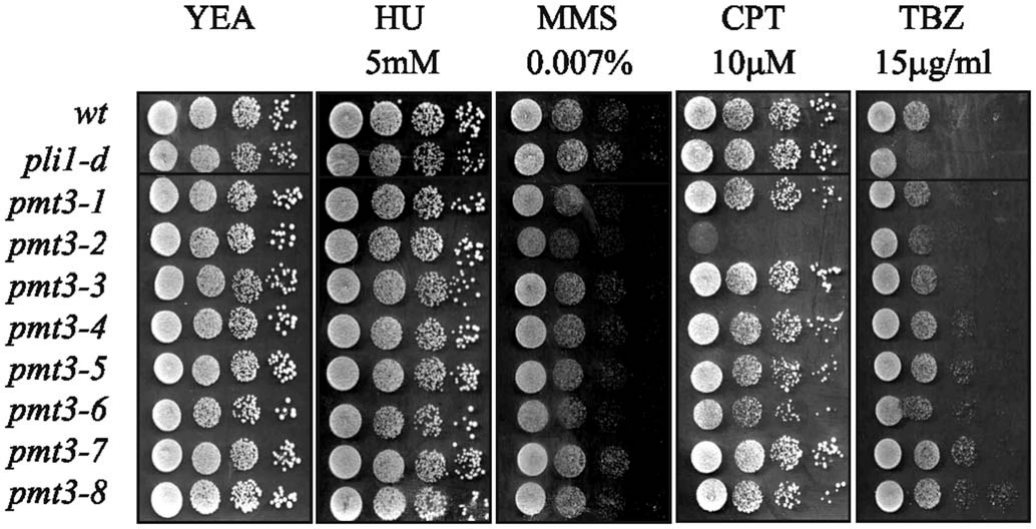
Effect of pmt3 mutations on sensitivity to DNA damaging agents, HU and microtubule inhibitors. 10 µl of 10 fold serial dilutions were plated onto media as indicated, and incubated at 30°C for 5 days.

We were interested to determine whether inability to phosphorylate SUMO/Pmt3 affected the response of cells to DNA damaging agents and other stresses. *pmt3-1* is not sensitive to HU, MMS, CPT or TBZ (Figure 4) or UV, IR or 1M sorbitol (data not shown), despite having significantly reduced levels of high Mr SUMO-containing species and free SUMO/Pmt3. This lack of phenotype is reminiscent of *pli1-d* cells which also have little sensitivity to these agents, apart from slight sensitivity to TBZ (Figure 4 and [27])

### *pmt3-2* is sensitive to camptothecin and MMS

Analysis of the sensitivities of the remaining seven *pmt3* mutants, (*pmt3-2* – *pmt3-8*) (Figure 4) indicates that *pmt3-2* (*pmt3-S15A*) is sensitive to the topoisomerase I inhibitor, camptothecin (CPT), and slightly sensitive to MMS. The reason for this is not known but may reflect a requirement for S15 in a process required for replication of a damaged DNA template.

### Sequence requirements for SUMO chain formation

The N-termini of *S. cerevisiae* SUMO/Smt3 and human SUMO-2/3 contain lysine residues that are involved in SUMO chain formation. Specifically, mutational analysis indicates that the major branch sites used during SUMO chain formation are K15 and K11 in *S. cerevisiae* Smt3 and human SUMO-2/3 respectively [17], [19], [20]. These lysine residues both occur within the SUMO acceptor consensus motif, ψKxE. The N-terminal region of *S. pombe* SUMO/Pmt3 does not contain a KxE motif (Figure S1). This suggests that the sequence requirement(s) for SUMO chain formation may be less stringent in *S. pombe* than in other organisms. Instead, SUMO/Pmt3 contains the sequence DV**K**PST (aa 28–33), adjacent to the highly conserved region of the molecule, and which corresponds to EV**K**PET (aa 17–22) in *S. cerevisiae*. In addition to K30, SUMO/Pmt3 has another lysine residue (K14) in its N-terminus. We have previously shown that K30 can act as a SUMO acceptor for chain formation in *S. pombe* [26]. We also demonstrated that the SUMO ligase, Nse2, can enhance SUMO chain formation.

**Figure 5:**
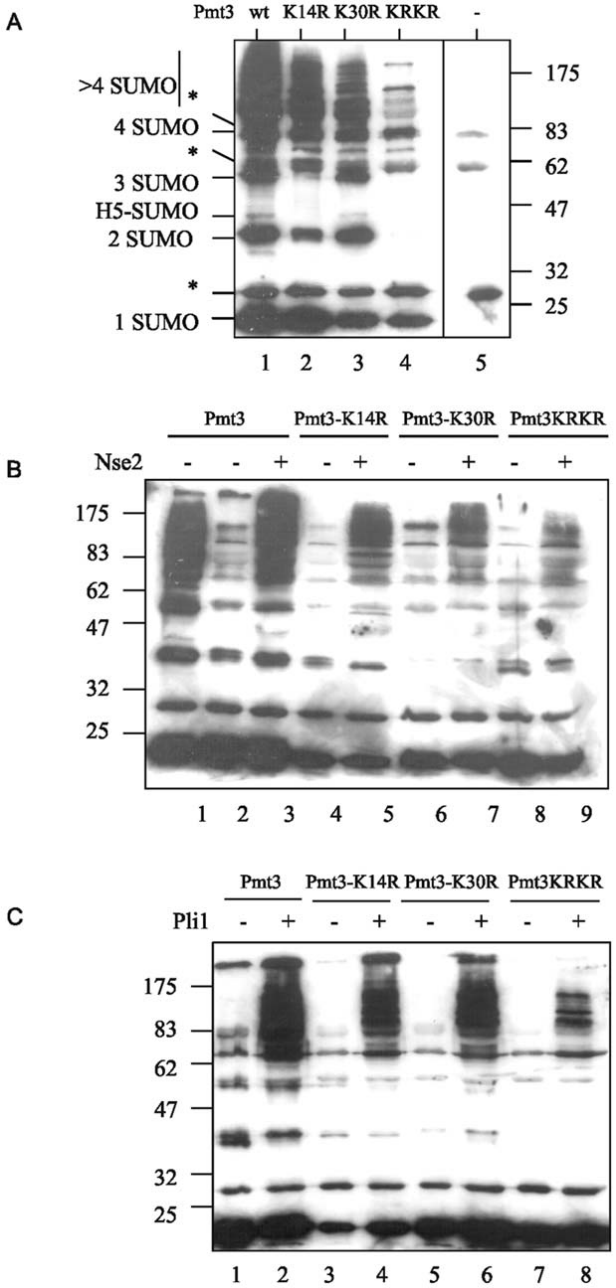
Pmt3 sequence requirements for chain formation. A–C. Mutant forms of Pmt3 tested for ability to form Pmt3 chains using the *in vitro* sumoylation assay in the absence of target protein. A. Lanes 1 Pmt3-GG, Lane 2 Pmt3-K14R,GG, Lane 3 Pmt3-K30R,GG, lane 4 Pmt3-K14R,K30R,GG, lane 5 no Pmt3. Reactions were carried out with 3 µg Hus5. B. Lane 1 3 µg Hus5, Lanes 2–9 0.3 µg Hus5, otherwise as indicated. C. Lanes 1–8 0.3 µg Hus5. * indicates cross-reaction with SAE proteins. H5-SUMO represents sumoylated Hus5.

We were interested in whether K14 can also be used as a SUMO acceptor, and whether the other *S. pombe* SUMO ligase, Pli1, can facilitate SUMO chain formation. Figure 5A indicates that under our standard *in vitro* SUMO modification conditions in the absence of either of the SUMO ligases, Nse2 or Pli1, mutation of lysine 14 to arginine (Pmt3-K14R) (lane 2), results in a similar decrease in SUMO chain formation as is observed with Pmt3-K30R (lane 3) when compared to wild type SUMO/Pmt3 (lane 1). Replacement of both lysine residues with arginine (Pmt3-K14R,K30R) further decreases chain formation (lane 4). These results indicate that both K14 and K30 act as SUMO acceptors *in vitro*, and that it is unlikely that there are other lysine residues within SUMO/Pmt3 involved in chain formation.

We next compared the effect of the K14R, K30R and K14R+K30R mutations on chain formation facilitated by the two SUMO ligases. Figure 5B shows that, as previously described [26], using Nse2 as a ligase, chain formation is reduced with Pmt3-K30R (lane 7) when compared to wild type Pmt3 (lane 3). Using Pmt3-K14R (lane 5) chain formation is also reduced, but to a somewhat lesser extent than with Pmt3-K30R (lane 7). Mutation of both lysines (Pmt3-K14R,K30R) essentially abolishes chain formation (lane 9). When Pli1 is used as a ligase, there is only a small reduction in chain formation with either Pmt3-K14R (Figure 5C, lane 4) or Pmt3-K30R (lane 6) as compared to wild type Pmt3 (lane 2). As is observed with Nse2, mutation of both lysine residues (Pmt3-K14R,K30R, lane 8) abolishes chain formation. Taken together, these results show that both K14 and K30 can act as SUMO acceptor sites *in vitro* when chain formation is facilitated by either of the SUMO ligases. Since mutation of both residues abolishes chain formation, it is likely that K14 and K30 are the only SUMO acceptor sites in SUMO/Pmt3.

### Inability to form SUMO chains results in aberrant cellular morphology and sensitivity to hydroxyurea

**Figure 6:**
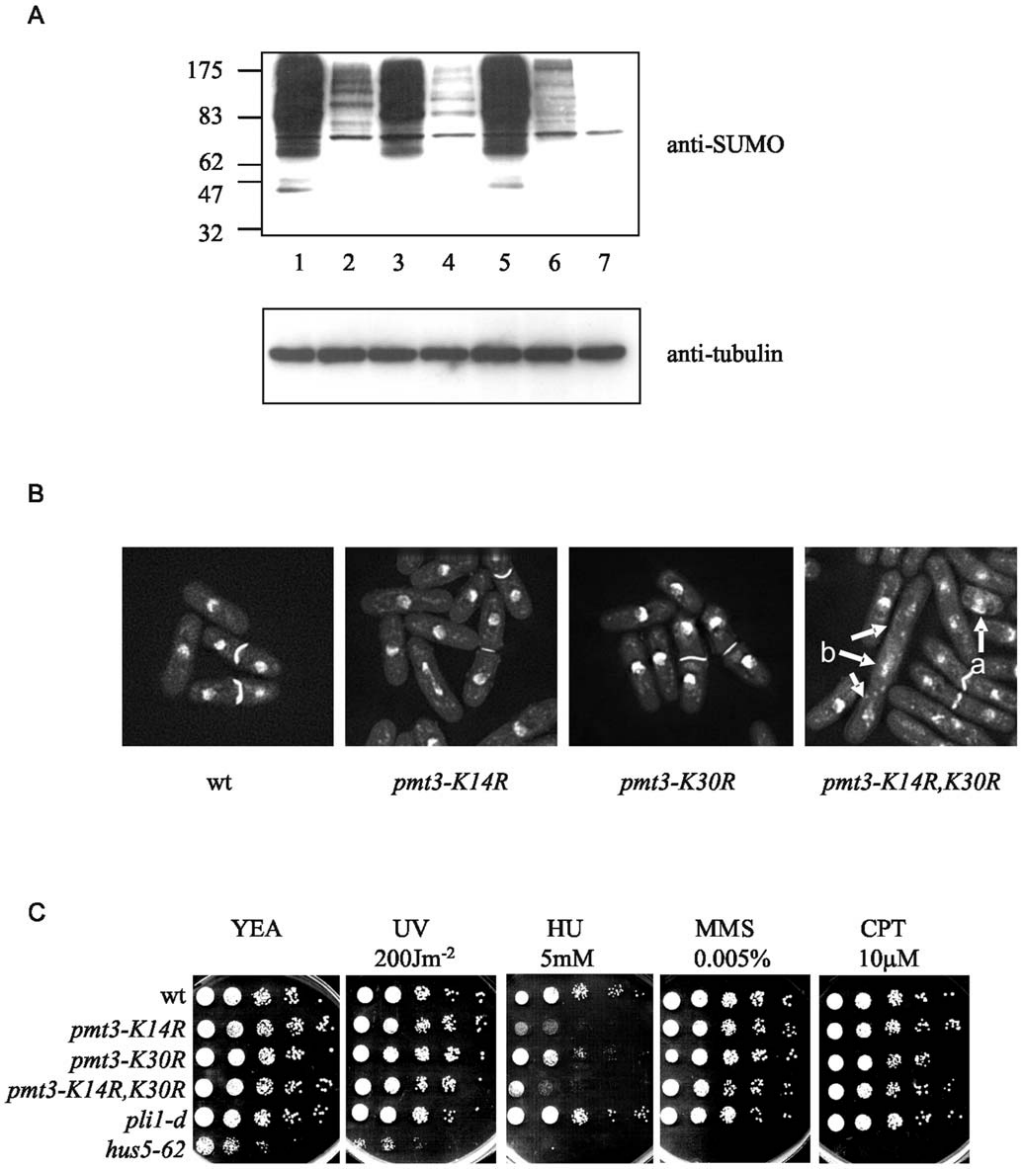
Phenotype of mutants defective in SUMO/Pmt3 chain formation. A. Western analysis of total cell extracts from cells containing mutant versions of SUMO/Pmt3. Lane 1,5 wt, lane 2 *pmt3-K14R*, lane 3 *pmt3-K30R*, lane 4 *pmt3-K14R,K30R*, lane 6 *pli1-d*, lane 7 *hus5-62*. B. Morphology of methanol fixed cells, stained with DAPI and calcofluor. C. Phenotype of *pmt3* mutants. 10 µl of 10 fold serially diluted cultures were plated onto media as indicated, and incubated at 25°C for 5 days.

We were next interested in determining whether any of the *pmt3* K to R mutations affect SUMO modification or chain formation when the mutant alleles are present in cells as the sole copy of SUMO/Pmt3 and whether they affect cell viability or morphology. All three mutants are viable, although *pmt3-K14R,K30R* colonies grow slightly slower than wild type (data not shown). Western blotting with anti-SUMO antisera indicates that, compared to wild type, *pmt3-K14R* has substantially reduced levels of high Mr SUMO-containing species (Figure 6A, lanes 2), compared to wild type cells (lanes 1,5). Cells containing *pmt3-K30R* (lane 3) have a similar level of high Mr SUMO/Pmt3-containing species to that observed in wild type cells (lanes 1,5), but the double mutant *pmt3-K14R,K30R* has significantly reduced levels high Mr species (lane 4), intermediate between those observed in *pli1-d* and *hus5-62* cells (defective in the SUMO conjugator, [24], [25]). These data show that K14 and, to a lesser extent, K30 are required for SUMO chain formation *in vivo*.

Mutants defective in sumoylation e.g. *rad31-d* (deleted for one subunit of the SUMO activator [23]) and *hus5-62* have aberrant cell and nuclear morphologies under normal growth conditions, and are sensitive to DNA damaging agents and the DNA synthesis inhibitor hydroxyurea (HU) [24], [25]. Comparison of the morphologies of *pmt3-K14R* and *pmt3-K30R* with that of wild type cells, indicates that replacement of a single lysine residue has no effect on cell or nuclear morphology, as cells resemble wild type (Figure 6B). However *pmt3-K14R,K30R* cells display a range of cellular morphologies, including elongated cells, aberrant nuclear structure (labelled a) and stretched and fragmented chromatin (labelled b). This phenotype is reminiscent of the phenotypes of *rad31-d* and *hus5-62* cells [23], [24], [25] and indicates that SUMO chain formation is important for normal growth under vegetative conditions.

We next investigated whether any of these *pmt3* mutants were sensitive to HU or other toxins. All the SUMO chain mutants resemble wild type in their response to UV, MMS, CPT and TBZ (Figure 6C and data not shown). The *pmt3-K14R* and *pmt3-K14R,K30R* mutants are both sensitive to HU (5 mM), while *pmt3-K30R* is very slightly sensitive to HU. These data indicate that K14, and possibly K30, is required for response of cells to replication arrest. The HU sensitivities of *pmt3-K14R* and *pmt3-K14R,K30R* are significantly less than that observed for *hus5-62*, indicating that as well as a requirement for SUMO chains, modification of target proteins by single SUMO/Pmt3 moieties is also likely to be necessary for the response to S phase arrest.

## Discussion

We show here that SUMO/Pmt3 is phosphorylated at its extreme N-terminus. While this manuscript was in preparation, results from mass spectrometry studies were published [29] which indicate that human SUMO-1 and *S. cerevisiae* SUMO/Smt3 are phosphorylated on S2. Our results described here, which show that *S. pombe* SUMO/Pmt3 is likely phosphorylated on two of three serine residues, S2, S4 and S6 (or diphosphorylated on one of them), are consistent with these data. [29]. In addition to being phosphorylated, Matic et al. observed that SUMO is acetylated on its N-terminus. Acetylation of SUMO/Pmt3 would account for the species labelled # (Figures 1 and 2) that remains after treatment with CIP.

Interestingly, inability to phosphorylate SUMO/Pmt3 in *pmt3-1*, results in a reduction in the level of high Mr SUMO containing species. Despite this, *pmt3-1* cells grow as wild type and are not sensitive to DNA damaging agents, HU or TBZ. Another feature of *pmt3-1* cells is the reduced level of free SUMO/Pmt3 (Figure 3B) suggesting that phosphorylation of SUMO/Pmt3 is required for stability of the molecule. The fact that *pli1-d* cells have normal levels of free SUMO/Pmt3 indicates that the low level of free SUMO/Pmt3 in *pmt3-1* is not likely to be due to the fact that in these cells, it is inefficiently conjugated onto target molecules.

It is now well documented that SUMO is capable of forming chains [17], [18], [20], [30] and reviewed in [31]. We show here that *S. pombe* SUMO/Pmt3 can form chains using two lysine residues in the N-terminus (K14 and K30). Interestingly, the K30R mutation has a somewhat greater effect on Nse2-dependent chain formation [26] than it does on Pli1-dependent chain formation (Figure 5B,C). The reason for this is not known, i.e. whether it reflects the fact that the two SUMO ligases have different specificities, or whether Pli1 behaves in a more promiscuous manner and can select K14 as an acceptor site if K30 is mutated. The sensitivities of the three SUMO chain mutants to HU, but not to a range of other genotoxins indicates a role for SUMO chains in the cell's response to S phase arrest. The fact that *pmt3-K14R,K30R* cells have aberrant cell and nuclear morphologies, while the two single mutants appear morphologically wild type indicates a role for both lysine residues. The morphology of *pmt3-K14R,K30R* is reminiscent of *rad31-d* and *hus5-62* mutants [23], [24], [25]. These results are in contrast to what has been observed in *S. cerevisiae*, where an *smt3-allR* mutation has no effect on vegetative growth or sensitivity to toxins [20].

It has recently been shown that SUMO chains interact with STUbLs, and can be ubiquitinated by them [21]. If one of the functions of chain formation is to facilitate the interaction of SUMO/Pmt3 with the STUbLs it might be expected that the phenotype of a mutant defective in chain formation would share similarities with the phenotypes of STUbL mutants. In *S. pombe* the STUbL proteins include Slx8, Rfp1 and Rfp2 [14], [32]. Deletion of *slx8* or of both *rfp1* and *rfp2* is lethal, while a conditional mutant of *slx8* (*slx8-1*) is sensitive to HU, MMS and CPT [14]. *pmt3-K14R,K30R* has a similar sensitivity to HU as *slx8-1*, but is wild type for response to MMS and CPT. The reason for the difference in MMS and CPT sensitivity between *slx8-1* and the SUMO chain mutants could be explained if the *S. pombe* STUbLs do not necessarily need to interact with SUMO chains in order to be targeted to substrates, but could recognise single SUMO species. This would be consistent with the fact that unlike the human STUbL, RNF4, that contains multiple SIMs which are proposed to recognise SUMO chains [21], *S. pombe* STUbLs only contain one or two SIMs [14], [32], suggesting that they interact with mono-sumoylated species.

In summary, the N-terminus of SUMO/Pmt3 is required for the formation of SUMO chains and is phosphorylated. Surprisingly, a *pmt3* allele encoding a non-phosphorylatable version of SUMO/Pmt3 behaves as wild type. In contrast, abolition of SUMO chain formation has a substantial effect on cell and nuclear morphology. In particular, SUMO chain formation is required for a process associated with S phase arrest, perhaps involving the STUbLs. The precise role of SUMO chains in this event i.e. the identity of protein(s) required for the response to S phase arrest, that are modified by SUMO chains remains to be determined.

## Materials and Methods

### Strains and plasmids

Strains were constructed using standard genetic techniques. The *pli1-d* mutant was created by deleting the ORF using the method of Bahler *et al*. [33], *hus5-62* was from A. Carr, Sussex [24]. Full length *pmt3* and *pmt3-GG* were amplified as described in [28]. *pmt3-nfl*, lacking the coding sequence for 29 aa at the N terminus, was produced by PCR. *pmt3* mutant alleles were produced by Quikchange PCR mutagenesis (Stratagene) according to the manufacturer's instructions. Mutant *pmt3* alleles were subsequently subcloned into pET15b (Novagen) for expression in *E. coli*, or integrated into the *S. pombe* genome along with 0.5 kb 5′ and 3′ *pmt3* flanking sequences and the *ura4* gene as selectable marker.

### Protein and Immunological methods

Whole cell *S. pombe* extracts were prepared using TCA as described in [34]. 1D SDS PAGE and Western blotting was carried out as described in [35]. 2D PAGE was undertaken using standard techniques [36]. Total cell protein from 10 OD_595_ units of cells were precipitated with TCA. The precipitate was resuspended in ice-cold acetone and protein precipitated at 4°C for 30 min. The precipitate was harvested by centrifugation at 13 krpm for 30 min, and allowed to dry. The pellet was resuspended in rehydration buffer (9M urea, 4% CHAPS, 2% IPG buffer (25 µl/µg pellet). DTT was added to 0.5%. 50 µg protein was added to modified rehydration buffer (6M urea, 2M thiourea, 2% CHAPS, 2% IPG buffer) to produce a total volume of 125 µl. 7 cm IPG strips pH 3–6 were used for the first dimension. 12.5% acrylamide was used for the second dimension. Anti-SUMO/Pmt3 antiserum was produced against full length SUMO/Pmt3 using standard methods [35]. Western analysis using purified recombinant proteins indicates that the antisera recognises equally well full length SUMO/Pmt3, N-terminally truncated SUMO/Pmt3 (Pmt3-nfl), Pmt3-1, and Pmt3 K to R mutants (Figure S3). Monoclonal anti-tubulin antibodies were from Sigma. The *in vitro* sumoylation assay was used as described previously [28].

### Phenotypic analysis of mutants

Sensitivities to hydroxyurea (HU), methyl methanesulphonate (MMS), camptothecin, (CPT) and thiabendazole (TBZ) were analysed on YEP agar at the doses stated. Plates were incubated at 30°C or 25°C for 5 days as stated. Cell morphology was analysed using methanol-fixed cells stained with DAPI (1 µg/ml) and calcofluor (0.5 µg/ml) using an Applied Precision Deltavision Spectris microscope.

## Supporting Information

Figure S1 SUMO sequence alignments Comparison of *S. pombe* (Sp), *S. cerevisiae* (Sc), human (Hs), *D. melanogaster* (Dm) and *C. elegans* (Ce) SUMO sequences, created using the ClustalW program. * indicates K14 and K30
(9.49 MB TIF)

Figure S2 2D PAGE of total *S. pombe* proteins. 50 µg of a wild-type total cell extract was separated using IEF strip pH 3–6, followed by SDS PAGE (12.5% acrylamide). Gel was stained with colloidal Coomassie Blue.
(9.29 MB TIF)

Figure S3 Anti-SUMO antisera recognise wt and mutant SUMO/Pmt3 Coomassie Brilliant Blue (CBB) staining and western analysis of recombinant wild type and mutant SUMO/Pmt3 with anti-SUMO antisera.
(9.42 MB TIF)
